# *Campylobacter jejuni* virulence factors: update on emerging issues and trends

**DOI:** 10.1186/s12929-024-01033-6

**Published:** 2024-05-01

**Authors:** Alexandra Tikhomirova, Emmylee R. McNabb, Luca Petterlin, Georgia L. Bellamy, Kyaw H. Lin, Christopher A. Santoso, Ella S. Daye, Fatimah M. Alhaddad, Kah Peng Lee, Anna Roujeinikova

**Affiliations:** 1 Biomedicine Discovery Institute, Department of Microbiology, Monash University, Melbourne, VIC 3800 Australia; 2 Department of Biochemistry and Molecular Biology, Monash University, Melbourne, VIC 3800 Australia

**Keywords:** *Campylobacter jejuni*, Pathogenesis, Biofilm, Chemotaxis, Polysaccharide capsule, Vaccine

## Abstract

*Campylobacter jejuni* is a very common cause of gastroenteritis, and is frequently transmitted to humans through contaminated food products or water. Importantly, *C. jejuni* infections have a range of short- and long-term sequelae such as irritable bowel syndrome and Guillain Barre syndrome. *C. jejuni* triggers disease by employing a range of molecular strategies which enable it to colonise the gut, invade the epithelium, persist intracellularly and avoid detection by the host immune response. The objective of this review is to explore and summarise recent advances in the understanding of the *C. jejuni* molecular factors involved in colonisation, invasion of cells, collective quorum sensing-mediated behaviours and persistence. Understanding the mechanisms that underpin the pathogenicity of *C. jejuni* will enable future development of effective preventative approaches and vaccines against this pathogen.

## Background

*Campylobacter jejuni* is an important human pathogen regarded as a major causative agent of bacterial gastroenteritis. *C. jejuni* infection typically causes self-limiting mild to severe diarrheal symptoms, accompanied by fever, nausea and abdominal cramping, which usually last about a week [1]. However, cases of invasive disease such as bacteraemia [2] have also been attributed to *C. jejuni* infection. In addition, *C. jejuni* gastroenteritis (campylobacteriosis) has been associated with myocarditis [3], as well as with many long-term sequalae, including dysbiosis, irritable bowel syndrome (IBS), and Guillain-Barré syndrome (GBS). *C. jejuni* gastroenteritis is a zoonotic disease: *C. jejuni* is transmitted to humans from animals, primarily chickens, where *C. jejuni* is part of normal intestinal microbiota, but can also be transmitted from cattle, pigs, sheep and, as the more recent evidence suggests, domestic cats and dogs [4]. *C. jejuni* infection in humans is typically acquired through contact with contaminated animal food products, most commonly poultry, as well as through contaminated water and milk [5].

**Fig. 1:**
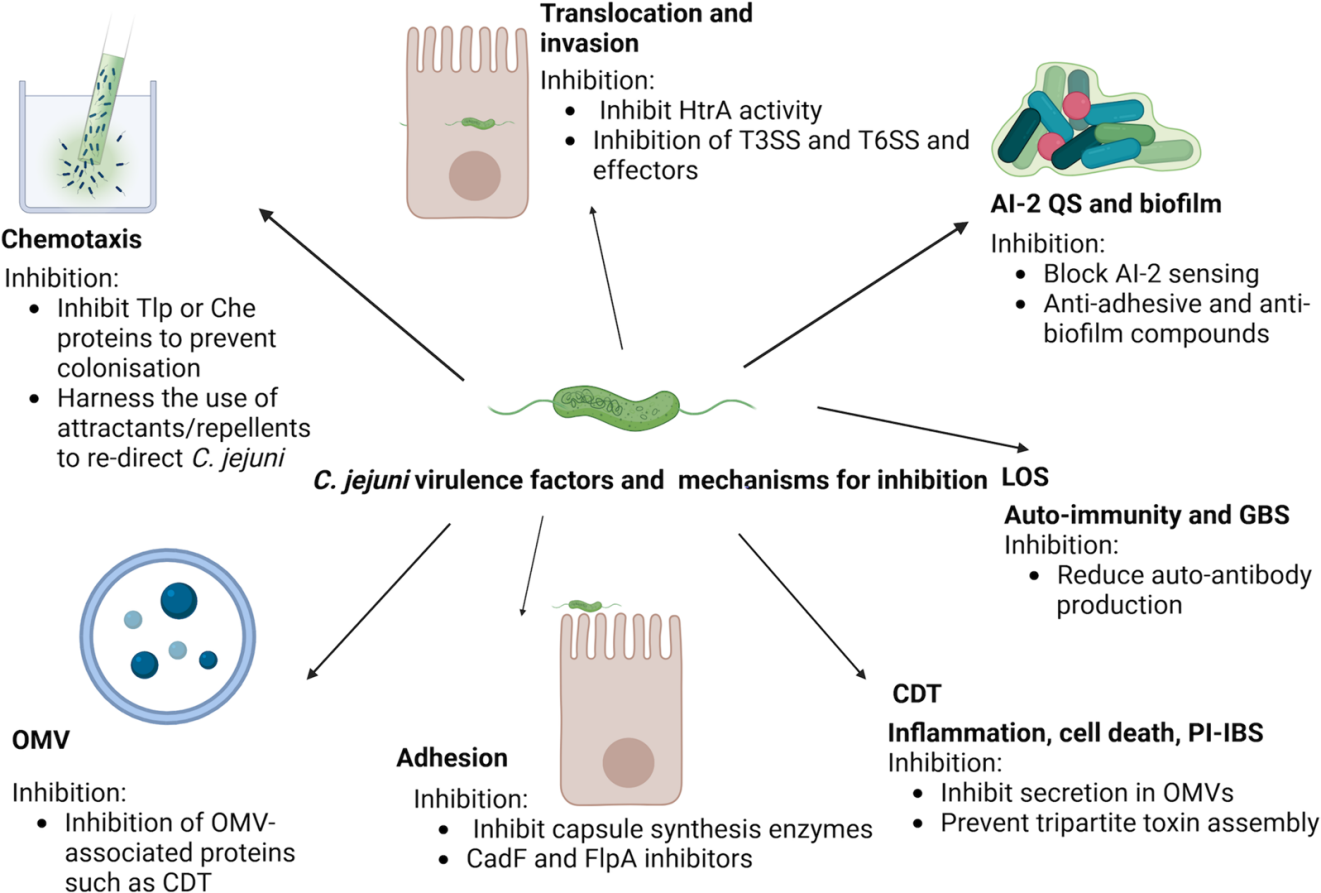
*C. jejuni* virulence mechanisms discussed in this review and potential strategies for their inhibition. Chemotaxis, facilitated by Transducer-like proteins (Tlps) and Chemotaxis (Che) proteins, enables *C. jejuni* to navigate towards favourable environments for colonisation. Targeting Tlp/Che signaling offers therapeutic potential. While the mechanisms of translocation and invasion of *C. jejuni* are not fully understood, the protease HtrA has been implicated in mediating paracellular translocation. In addition, type III and type VI secretions system (T3SS, T6SS) have been identified to as key players in invasion, with the T3SS effector CiaI facilitating intracellular survival. Inhibiting HtrA presents a promising strategy to diminish translocation and invasion. Impeding the assembly of T3SS or T6SS, or inactivating their effectors, could be an innovative strategy for future therapeutic interventions. The role of biofilm formation in the environmental survival of *C. jejuni* is well recognised. While further investigation into the role of quorum sensing and biofilm formation in human campylobacteriosis is still needed, targeting AI-2 sensing and biofilm formation as a way to reduce persistence should be explored in future research. Adhesion is an important virulence mechanism facilitating colonisation and invasion. Development of anti-adhesive agents or blockers of known adhesins such as CadF or FlpA represents a novel therapeutic direction. As the capsule plays a role in adhesion, inhibiting capsule biosynthesis enzymes may reduce virulence, as well as potentially reduce immune evasion. Recent research highlights the complexity, phase variation and diversity of capsule biosynthesis pathways, making this one of the central topics for future *C. jejuni* investigations. The cytolethal distending toxin (CDT) is a major pro-inflammatory and cytotoxic agent of *C. jejuni*. Preventing either its secretion in outer membrane vesicles (OMVs) or its assembly into a tripartite holotoxin could attenuate *C. jejuni* virulence. Identifying other OMV-secreted proteins and blocking their action presents another anti-virulence strategy. Recent research into anti-OMV vaccine has shown promise and warrants further investigation

Recent advances in the application of genomics, transcriptomics, proteomics and cutting-edge biochemical analysis to the study of *C. jejuni* pathogenesis have significantly enhanced the understanding of the molecular mechanisms that underpin disease development and bacterial persistence, both in the host-related environment, and in environments associated with food processing. This review explores the latest major findings in this field, highlighting discoveries pertaining to each key factor in *C. jejuni* infection and persistence, as well as progress in *C. jejuni* vaccine studies. A summary of the central virulence factors discussed in this review, as well as potential approaches for inhibiting these virulence mechanisms, are presented in Fig. 1.

### Motility and colonisation

*C. jejuni* must be able to penetrate the highly viscous mucous layer of the gastrointestinal tract to reach the epithelium and colonise the intestinal crypts [6]. Motility is, therefore, one of the key virulence factors required for colonisation in both animal and human hosts [7–9]. *C. jejuni* has a spiral-shaped cell body with a single flagellum at both poles. The spiral shape [6] and the rotation of the flagella function to propel the bacteria in a corkscrew-like motion that confers an advantage to move in highly viscous environments such as mucus [10]. The leading flagellum of the swimming cell was found to wrap around the helical cell body, with the lagging flagellum rotating freely, although the wrapped flagellum also rotates and contributes to propulsion [10]. The movement of flagella is controlled by the sensing of extracellular stimuli in a process termed chemotaxis, which enables *C. jejuni* to swim towards or away from the stimuli [11]. In addition to mediating motility, the flagella of *C. jejuni* act as a type III secretion system (T3SS) to enable the secretion of the flagellum components during its self-assembly and of bacterial proteins into the host cytosol during the invasion process [12].

Chemotaxis (directed motility) enables *C. jejuni* to move towards favourable conditions or away from detrimental ones, resulting in bacterial colonisation of optimal niches. To sense environmental stimuli, *C. jejuni* uses chemoreceptors, also known as transducer-like proteins (Tlps). Tlps may be transmembrane or cytoplasmic proteins, which, in response to a stimulus, initiate signaling through a two-component system. *C. jejuni* is thought to possess more than 10 Tlps, the cognate signals for some of which have been identified and include various components in the mucus layer such as mucin, amino acids, and organic acids and glycans [11, 13, 14].

The two-component chemotaxis system in *C. jejuni* consists of histidine kinase protein (CheAY), and a response regulator (CheY). Several accessory proteins are also involved in the sensing of chemoeffectors by this system, including the adaptor proteins (CheW, CheV) and the methylation adaptation enzymes (CheR, CheB) [15]. Upon activation of the sensory domain of Tlps by a chemorepellent (or in the absence of attractant), CheAY will autophosphorylate and subsequently phosphorylate CheY [16]. The phosphorylated form of CheY binds to the switch complex of the flagellar motor, resulting in a change from a counterclockwise to a clockwise flagellar rotation [15, 17]. In the case of attractant binding, CheAY binds to the adaptor protein CheV, inhibiting the autophosphorylation of CheAY, resulting in reduced phosphorylation of CheY and no binding to the switch complex. This results in counterclockwise rotation. The adaptor proteins (CheW or CheV) assist in the binding of CheAY to the chemoreceptor, and alter autophosphorylation of CheA [18]. Recently, knockout strains of *cheW* and *cheV* have both demonstrated a decrease in chemotactic motility on soft agar plates, with the *cheV* mutant showing more drastic reduction than the *cheW* mutant [15]. CheR and CheB mediate adaptation to the chemotaxis signal, whereby CheR methylates specific glutamyl residues on chemoreceptors, stimulating CheAY autophosphorylation in the presence of an attractant, which results in clockwise flagella rotation [17, 18]. CheB demethylates these residues in the presence of a repellent, restoring rotation to counterclockwise [17, 18].

While significant progress has been made in identifying physiologically relevant attractants and repellents in *C. jejuni* chemotaxis (reviewed in [19]), it remains poorly understood how multiple signals from the more than 10 Tlps are integrated in a complex in vivo environment, where various combinations of attractants and/or repellents may be present. Furthermore, little is known about how these signals integrate with other virulence mechanisms, such as biofilm formation, for example. In addition, while it is recognised that vaccines or therapeutics targeting *C. jejuni* chemotaxis may prevent colonisation or reduce its persistence in the host, limited work has been done to develop such intervention approaches.

### Translocation to the basolateral surface of the epithelium

**Fig. 2:**
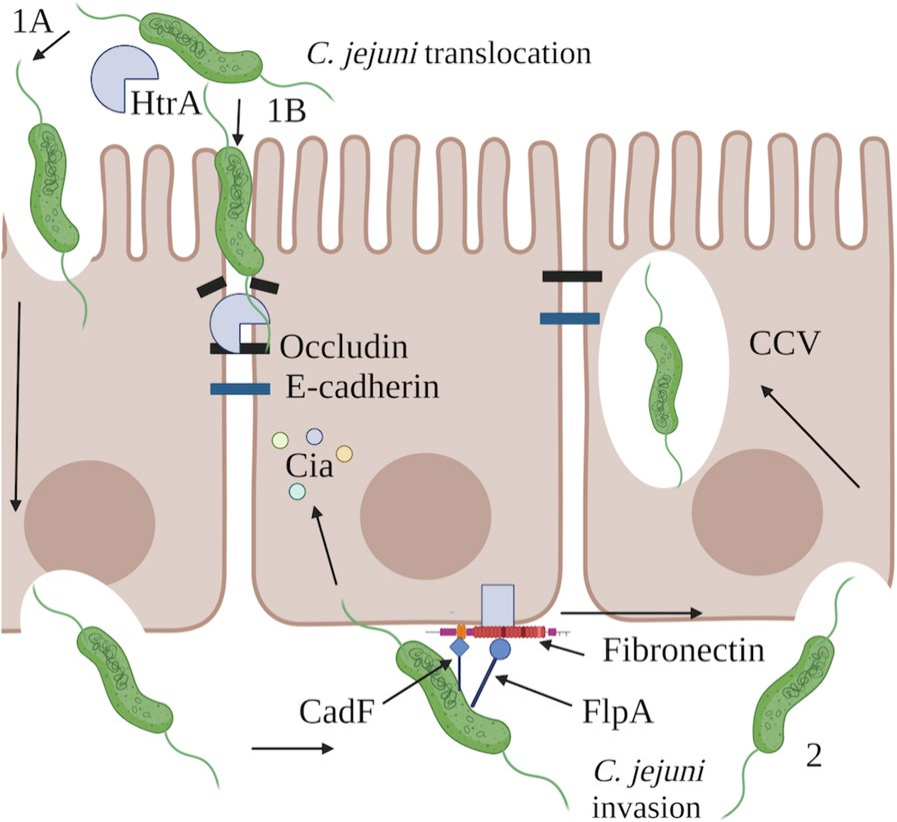
The proposed translocation and invasion mechanisms utilised by *C. jejuni.*1A. *C. jejuni*translocation by the transcellular route, whereby *C. jejuni* enter the intestinal epithelial cells at the apical side, and are able to transmigrate to the basolateral side. 1B. *C. jejuni*translocation by the paracellular route, whereby *C. jejuni* secretes HtrA, which is able to cleave occludin at tight junctions of epithelial cells, and E-cadherin at adherens junctions of epithelial cells. HtrA secretion disrupts the barrier between epithelial cells and allows *C. jejuni* passage between epithelial cells to the basolateral cell surface. From here, *C. jejuni* is able to initiate invasion. 2.*C. jejuni* invasion of intestinal epithelial cells. Invasion is mediated by injection of the Cia effector proteins into the host cell cytosol. Additionally, invasion is initiated by the binding of fibronectin by *C. jejuni* proteins CadF and FlpA. This initiates a host-cell signalling cascade and initiation of cytoskeletal rearrangements, which enable uptake of *C. jejuni* intracellularly, into a Campylobacter Containing Vacuole (CCV). With the assistance of some of the Cia antigens, *C. jejuni* prevents the fusion of the CCV with lysosomes, and is able to persist intracellularly (figure modified and expanded from [23])

Translocation enables *C. jejuni* to migrate from the apical cell surface to the basolateral cell surface [20]. Access to the basolateral cell surface provides *C. jejuni* with a capacity for adherence to fibronectin and subsequent invasion of epithelial cells, a process which has recently been reviewed in depth [21], and a proposed summary of *C. jejuni* translocation mechanisms and subsequent invasion following basolateral fibronectin binding is shown in Fig. 2. Translocation to the underlying tissues has also been proposed be advantageous to *C. jejuni* by providing access to nutrients such as iron, and by preventing its clearance by the peristaltic forces of the intestine, thereby facilitating its persistence [22].

The route of *C. jejuni* translocation has been debated [22]. Early studies suggested a transcellular route that involves pathogenic bacteria entering host cells at the apical surface, intracellular trafficking across the cytoplasm, and the pathogen exit from the cell at the basolateral surface [24]. In contrast, more recent studies suggested a paracellular route of translocation for *C. jejuni*, which involves pathogenic bacteria travelling between adjacent epithelial cells through tight and adherens junctions [25]. For example, characterisation of serine protease HtrA (high-temperature requirement protein A) secreted by *C. jejuni* showed that HtrA can cleave E-cadherin - a component of tight junctions - enabling *C. jejuni* to cross a polarized MKN-28 cell layer via the paracellular route [23, 26]. Another recent study showed that when polarized intestinal Caco-2 cells were infected with *C. jejuni*, the tight junction protein occludin was cleaved by HtrA, resulting in its redistribution from the tight junctions into the cytoplasm [27]. Invasion was also enhanced in the occludin knockout cells, indicating enhanced *C. jejuni* penetration through disrupted tight junctions to reach basal membranes, and subsequently invade [27]. Importantly, the study detected a similar redistribution of occludin and the tight junction lateral plasma protein ZO-1 in the biopsy samples from campylobacteriosis patients, confirming disruption of tight junctions consistent with a paracellular translocation route of *C. jejuni* during infection [27]. Importantly, *C. jejuni* was recently shown to induce translocation of non-invasive *Escherichia coli* and *Lactococcus lactis* into the basal compartment in an HtrA-dependent manner [28], implicating *C. jejuni* HtrA in the pathogenesis associated with undesired immune responses to the intestinal microbiome.

### *C. jejuni* invasion of host cells

*C. jejuni* displays a facultatively intracellular lifestyle, which enables it to persist within intestinal epithelial cells, in specialised *Campylobacter*-containing vacuoles (CCVs), which do not fuse with lysosomes [29]. CCVs display the cellular markers of early to late endosomes (early endosomal antigen-1, GTPases Rab5 and Rab7, and lysosomal associated membrane protein 1), and do not associate with the lysosomal marker cathepsin [30]. One bacterial protein, the T3SS effector CiaI, has been shown to contribute to the mechanism by which the vacuole properties are altered. In HeLa cells infected with the *ciaI* mutant, 80% of CCVs were found to associate with cathepsin, whereas such an association was found to occur in only 20% of the wild-type (WT) CCVs [30]. While the reason for CiaI preventing fusion with lysosomes was not established, it has been proposed that this could occur as a result of either preventing acquisition of a host cell protein essential for vacuole maturation, retention of markers which may inhibit vacuole maturation, or selective acquisition of a marker not usually associated with the maturation process [30]. Interestingly, this phenomenon of CCVs avoiding fusion with lysosomes appears to be host-cell specific, as it only occurs in intestinal epithelial cells, and does not occur when *C. jejuni* invades macrophages. Within the CCVs, *C. jejuni* is able to reside in a dormant, viable but non culturable cell state, and this plays a role in its evasion of the immune response [29]. Although it has been known for a while that *C. jejuni* is able to invade colonic epithelial cells and goblet cells during infection [31], the mechanisms of how *C. jejuni* invades host cells and persists intracellularly are not yet completely understood.

Generally, bacterial invasion of host cells can occur by 2 distinct mechanisms known as the “zipper” and “trigger” routes [23]. The zipper invasion mechanism involves binding of the bacterial adhesin to specific receptors on the host cell, followed by subsequent cytoskeletal rearrangements to engulf the bacterium via endocytosis. The trigger invasion mechanism involves a T3SS-mediated injection of the pathogen’s effector proteins into the cytosol of the host cell, which triggers a signalling cascade resulting in membrane ruffling and bacterial uptake in a macropinocytosis-like manner [23, 32]. However, the invasion mechanism for *C. jejuni* may be unique in a sense that it appears to have elements of both the zipper and trigger mechanisms [23, 33].

A vast array of *C. jejuni* cell surface molecules were reported to facilitate both adhesion to, and invasion of intestinal epithelial cells. Adhesion permits initial association with host cells and prevents the removal of bacteria by the peristalsis-driven flow of gastric fluid [34]. Adhesion can subsequently facilitate interactions between the bacterial and host cells leading to the invasion of the host cells - for example, it may serve as a platform for the delivery of effector molecules by the T3SS [35].

Several *C. jejuni* proteins have been proposed to play a role in adhesion, including CadF, FlpA, JlpA, Peb1 and Peb3 [36]. Among these, FlpA and CadF are the most extensively studied ones. CadF and FlpA are able to bind fibronectin [37], a component of the extracellular matrix of intestinal epithelial cells, which is predominantly found at the basolateral surface. The roles of CadF and FlpA in host cell adhesion and invasion have been recently extensively reviewed [21]. It is believed that by binding to fibronectin at the basolateral cell surface, these proteins initiate a signalling cascade involving phosphorylation of paxillin, a central component of focal adhesions, as well as downstream signaling events that control membrane trafficking and cytoskeletal rearrangement aiding the invasion process [21]. Interestingly, adhesion via these proteins is likely to play a role in facilitating T3SS effector delivery, as evidenced by the reduced delivery of CiaC in a *flpA* mutant [38]. It is thought that attachment of *C. jejuni* to the host cell ensures close proximity necessary for Cia delivery into the host cell [39].

While invasion by other bacterial species is known to involve the host cell cytoskeleton proteins, the roles of actin-containing microfilaments or microtubulin-containing microtubules in *C. jejuni* invasion remain controversial [33], with some studies suggesting one or the other or both of the mechanisms [23, 33]. Remarkably, *C. jejuni* was shown to retain the ability to invade human intestinal Caco-2 cells following their treatment with ATP-depleting agent or microfilament- and microtubulin-inhibiting agents, suggesting that *C. jejuni* invasion may occur via a novel mechanism that does not require significant rearrangement of the cytoskeleton [40]. However, it was proposed that the degree of the cytoskeleton rearrangement during *C. jejuni* invasion may be host-dependent [41].

### *C. jejuni* secretion systems and invasion

Two secretion systems – the type III (T3SS), and type VI secretions system (T6SS) - have been identified in *C. jejuni*, and both have both been shown to play a role in its invasion of host cells. As mentioned above, the *C. jejuni* flagellum functions as a T3SS that facilitates the release of bacterial effector proteins. T3SS-mediated secretion of proteins known as *Campylobacter* invasion antigens (Cia) directly into the host cell cytosol triggers uptake of *C. jejuni* by the host cell [23]. The detailed mechanism of the Cia secretion is not fully understood. In one study, it was shown that expression of either flagellin FlaA or flagellin FlaB was required for the Cia secretion [42]. However, a different study found that while the flagellar hook was indispensable for the Cia secretion, the presence of FlaA and FlaB was not essential [39].

The first identified Cia effector protein, termed CiaB, comprises 610 amino acids and displays homology to T3SS effectors of other pathogens [43]. CiaB facilitates *C. jejuni* uptake by intestinal epithelial cells, as internalisation was significantly reduced in *ciaB* mutants. The presence of CiaB was required for the secretion of other Cia proteins. The secretion was induced by the contact with both viable intestinal epithelial cells and host cell lysates, suggesting that it is triggered by host-produced signal(s) [44]. CiaB function remains to be fully elucidated [45]. CiaB has been found in most *C. jejuni* isolates obtained from retail meats, despite the strains displaying variability in adherence and invasion capacities [46]. Moreover, there has been a controversial report of a negative association of CiaB with invasiveness, suggesting that the invasiveness of *C. jejuni* is determined by a complex interplay of Cia effectors and other proteins [47].

CiaC was identified using a genetic screen for sequences that contain an N-terminal T3SS secretion signal [48]. CiaC was shown to be secreted via the T3SS, and a *ciaC* mutant displayed reduced invasion of intestinal epithelial cells. It has been subsequently shown that CiaC induces host cell cytoskeletal rearrangements responsible for membrane ruffling and internalization of the bacteria [39]. CiaD was also confirmed to be secreted, and while its presence was not required for the secretion of other Cia proteins, it was shown to mediate invasion of human intestinal epithelial cells, and secretion of the inflammatory cytokine interleukin-8 (IL-8) [49]. Once in the cytosol of human intestinal epithelial cells, CiaD stimulates the mitogen activated kinase signaling pathways Erk 1/2 and p38 to promote host cell invasion and the release of IL-8 [49]. An independent mechanism of facilitating cell entry that does not involve the mitogen kinase domain of CiaD has also been described [50]. This effector has been shown to bind to a Ras GTPase-activating-like protein called IQGAP1 in the cytosol of the host cell, preventing the association of IQGAP1 with RacGAP1, and resulting in the unhindered activity of the Rho GTPase Rac1, which subsequently activates proteins WAVE2 and Arp2/3, leading to host cell actin reorganization, and consequently, *C. jejuni* internalization [50]. Furthermore, CiaD has been shown to be important for the generation of an inflammatory cytokine response by *C. jejuni* in a piglet ligated intestinal loop model [51]. Likewise, CiaD has been shown to play a role in disease development in a murine campylobacteriosis model [49, 51].

The final characterised Cia, CiaI, appears to enhance intracellular survival within epithelial cells [30]. CiaI harbours a dileucine motif which directs it to late endosomal vesicles, resulting in the prevention of the fusion of the CCVs with lysosomes [30]. Conversely, a different study concluded that it is the CiaI nucleotide binding domain that is important for facilitating invasion of human intestinal epithelial cells, whereas the dileucine motif appeared to be not essential [52]. Although the requirement for CiaI for invasion is well recognised, the precise molecular mechanism of its participation in this process remains to be elucidated. Interestingly, in addition to facilitating intracellular survival within human intestinal epithelial cells, CiaI has been shown to play a central role in commensal colonization of chicks [52].

It is important to note that, in addition to Cia proteins, the *C. jejuni* T3SS secretes many other effectors: a recent bioinformatic study of the common laboratory strains identified over 50 potential T3SS effector proteins, suggesting a more expansive role of this system in pathogenesis [45], however further studies are needed to unveil the function of these novel effector proteins.

In addition to the T3SS, a T6SS has also been found in *C. jejuni*. Interestingly, only ~ 16–20% of *C. jejuni* isolates have been shown to encode the T6SS [53, 54]. The *C. jejuni* T6SS has been shown to play a role in its adhesion and invasion of T84 colonic epithelial cells and murine macrophages, as well as in enabling colonisation in a murine model [53]. The T6SS has also been shown to mediate sensitivity to the bile salt deoxycholic acid, which drives bacteria to colonise the proximal colon, where the physiological levels of deoxycholic acid are lower than in the small intestine [53]. Interestingly, the T6SS was shown to facilitate the *C. jejuni* defense against oxidative stress, and to enhance colonisation in the chicken gut, which implies its involvement in commensalism in chickens, as well as pathogenicity in humans [55]. However, a clinical study did not find an association between the presence of T6SS and disease severity, although T6SS-positive strains were more prevalent in immunocompromised patients [56]. This highlights a need for more thorough investigations on the role of *C. jejuni* T6SS in colonisation and disease.

The hemolysin co-regulated protein (Hcp), known to play a role of both a structural protein and an effector protein of the T6SS [57] was shown to possess immunogenic potential in a chicken model [58]. Hcp also contributes to cytotoxicity towards erythrocytes, in a manner dependent on downregulation of capsule expression [59]. In addition, the T6SS was shown to play a major role in bacterial inter-species competition, whereby T6SS-positive strains of *C. jejuni* outcompeted *E. coli* within the same niche [60]. While the Hcp has been characterised well as both a structural and effector protein in *C. jejuni* T6SS [57], very little is known about the roles of other effectors of the *C. jejuni* T6SS and their specific functions during infection, although recent bioinformatic studies have identified several putative T6SS effectors, including nucleases and an NAD^+^-glycohydrolase [61, 62], which opens avenues for further investigation of their functions.

### Outer membrane vesicles

In addition to the T3SS and T6SS, *C. jejuni* secretes a wide milieu of proteins, including virulence factors, via outer membrane vesicles (OMVs). *C. jejuni* OMVs have been shown to harbour at least 150 different proteins, including outer membrane proteins (OMPs), periplasmic proteins, inner membrane proteins and cytoplasmic proteins [63]. Interestingly, all 3 subunits of the major *C. jejuni* toxin CDT has been found to be secreted via OMVs, and OMVs containing CDT induced cell distension and cell cycle arrest in HeLa cells [64, 65]. *C. jejuni* OMVs were shown to induce the secretion of IL-8, IL-6, TNF-α and the antimicrobial peptide hBD-3, in T84 cells [63]. Furthermore, *C. jejuni* OMVs were cytotoxic in a *Galleria mellonella* model, but in a manner not dependent on CDT presence, indicating that the cytotoxic effect was dependent on other proteins within the OMVs [63].

The protein composition of *C. jejuni* OMVs appears to be dynamic and influenced by environmental conditions (e.g. their change during colonisation of the host) [66]. The variation of the OMV proteome in different conditions suggests the importance of these proteins at different stages of infection and for adaptation to different hosts. Interestingly, OMVs were found to contain oxidoreductases involved in the TCA cycle, which may facilitate *C. jejuni* survival in the host, and flagellins and flagellar hook proteins, known to facilitate adhesion to host cells [66]. Temperature has also been shown to alter the OMV proteome, with distinct protein compositions at 37 °C (associated with the human host) and 42 °C (associated with the avian host). Significantly, OMVs produced at 37 °C had a higher abundance of virulence proteins compared to OMVs produced at 42 °C [67]. Furthermore, OMVs derived from *C. jejuni* 11168 and harbouring proteases HtrA, Cj0511 and Cj1365c contributed to the invasion of T84 monolayers due to the protease-dependent cleavage of both E-cadherin and occludin, although there remains a controversy regarding the ability of OMV-associated HtrA to disrupt cell junctions [68, 69].

Due to their importance in mediating interactions with the host, *C. jejuni* OMVs have recently been considered as a potential vaccine target. Intra-gastric immunisation of mice with purified chitosan-coated OMVs induced a mixed Th1/Th2 response, and significantly reduced the cecal load of *C. jejuni* upon challenge, indicating protective efficacy [70]. However, further studies are needed to determine the safety, considering the presence of LOS within the OMV.

### Polysaccharide capsule and immune evasion

The capsular polysaccharide (CPS) of *C. jejuni* forms an external layer on the cell surface, that contributes to *C. jejuni* virulence and immune evasion [71]. Indeed, an isogenic mutation in the *kpsM* gene, encoding a component of an ABC transporter involved in capsule biosynthesis, resulted in reduced adhesion to and invasion of human intestinal epithelial cells, reduced diarrhoea in a ferret model of disease [72], and impaired colonisation in a mouse model of infection [73]. Capsule-deficient mutants were also shown to have a reduced ability to colonise chickens [74].

Penner serotyping, also known as heat-stable (HS) serotyping [75], has been the approach used to classify *C. jejuni* into the 47 currently identified serotypes, where the capsule is the major serodeterminant. However, a PCR multiplex-based typing approach revealed that due to cross-reactivity [76], the number of capsule types represented by the 47 serotypes, is only 35 [77] (Table 1). The capsular biosynthesis pathway is complex and variable between strains [76], and the list of previously uncharacterised genes identified to be involved in capsular biosynthesis is expanding.

**Table 1:** List of 35 *C. jejuni* serotypes/cross-reactive serogroups as determined in [77]. Several capsule types that display high sequence identity of the capsule synthesis loci, are grouped into complexes which contain more than 1 serotype

Capsule type / complex	
HS1/44	Includes HS1, HS44
HS2	
HS3	
HS4 complex	Includes HS4/13/16/43/50/63/64/65
HS5/31	
HS6/7	Includes HS6, HS7
HS8/17	Includes HS8, HS17
HS9	
HS10	
HS11	
HS12	
HS15	
HS18	
HS19	
HS21	
HS22	
HS23/36	Includes HS23, HS36
HS27	
HS29	
HS32	
HS33	
HS35	
HS37	
HS38	
HS40	
HS41	
HS42	
HS45	
HS52	
HS53	
HS55	
HS57	
HS58	
HS60	
HS62	

The CPS is composed of repeating sugar units, and for some strains the CPS has been well characterised. For example, strain NCTC 11168, which belongs to the HS:2 serotype, has a CPS composed of repeating units of *D*-glycero-*L*-gluco-heptose, *D*-glucuronic acid, *D-N*-acetylgalactosamine, and *D*-ribose. Like strain 11168, the CPS of other *C. jejuni* strains frequently harbours heptose residues in unusual atomic configurations and spatial orientations such as “altro”, “gulo”, “gluco” and “ido” [78].

Strain-to-strain variation in the location of heptose sugars within the CPS has also been observed. For example, in strain 81–176, the heptose moiety is an inherent part of the repeating unit of the CPS, whereas strain 11168 possesses a heptose moiety that branches off from the repeating unit [79]. Because of this, the heptose synthesis mutants of strain 11168 still possess the CPS. Myles et al. [79] have recently taken advantage of this fact by using these mutants to elucidate the distinct roles of the CPS and its branching heptose during infection. The study found that the capsule reduced bacterial adherence to and uptake by chicken macrophages, and decreased intracellular survival, whereas the heptose moieties in the CPS had the opposite effect. Except for adherence, similar results were obtained for human macrophages. In addition, the heptose moieties were shown to reduce the capsule-induced nitrogen oxide (NO) generation by chicken macrophages. These findings highlighted the complex interplay of the capsule and its heptose moieties in *C. jejuni* 11168, suggesting that the CPS structure may undergo evolutionary adaptation to enhance bacterial survival in both commensal and pathogenic contexts [79].

Recent studies have enhanced our understanding of heptose biosynthesis central to the CPS. At least 10 different heptose sugars have been identified in different strains of *C. jejuni* [80]. GDP-*D*-glycero-α-*D*-manno-heptose has been identified as the starting point for the synthesis of all heptose moieties, and the 4 identified enzymes responsible for its synthesis have been found to be highly conserved across *C. jejuni* strains containing heptose sugars in the CPS [81].

The most extensively studied heptose moieties have been the 6-deoxy-heptoses. The first step in the synthesis of 6-deoxy-heptoses is the 4,6-dehydration of GDP-*D*-glycero-α-*D*-manno-heptose by a dehydratase [82]. In the second step, an epimerase racemises the C3 and/or C5 of the resulting GDP-6-deoxy-4-keto-*D*-lyxo-heptose. Of note, different serotypes possess epimerases that racemise either only the C3, or both C3 and C5 [83]. The last step is the reduction of the 4-keto-product by a C4-reductase [84]. To date, 20 enzymes with different substrate specificities have been identified in pathways involved in the synthesis of a range of heptose moieties in *C. jejuni* strains, and give rise to 6 variable 6-deoxy-heptoses, as well as other heptose variants present in the CPS [80].

C4 reductases in sequenced *C. jejuni* strains have been categorised into 9 clusters based on sequence similarity, with each cluster corresponding to the production of distinct forms of GDP-6-deoxy-heptose varying in stereochemistry [85]. Intriguingly, 2 phase variable C4-reductases were identified in gene clusters for the capsular polysaccharides in serotypes HS:10, HS:29, HS:41 and HS:63. One of the two was shown to be a bifunctional epimerase/reductase that can catalyse the epimerisation of C3 and C5 of GDP-6-deoxy-4-keto-*D*-lyxo-heptose before the reduction of C4. It was proposed that the phase variation of one of these enzymes could influence their expression levels and changes in the CPS structure [84].

The CPS of *C. jejuni* can be further modified by phosphoramidylation, methylation, as well as amidation with ethonolamine or serinol. The enzymes responsible for the amidation of the glucuronic acid component of the capsule in strain NCTC 11168 have recently been identified as a pyridoxal-5′-phosphate (PLP)-dependent transaminase, and a PLP-dependent decarboxylase [86].

Modifications of sugars by the addition of O-methyl phosphoramidate (MeOPN), have been observed in 68–80% of *C. jejuni* strains [87]. A study with strain 81–176, where MeOPN transferases can add MeOPN at 3 distinct positions of the galactose moiety of the CPS, identified the importance of MeOPN modifications in mediating resistance of *C. jejuni* to normal human serum [88]. This suggested that MeOPN may prevent binding of pre-existing antibodies to the CPS, and activation of the classical complement pathway, suggested to be the major pathway for clearance of *C. jejuni* [88]. The non-stoichiometric nature of MeOPN modifications by the 2 MeOPN transferases in strain 81–176 has been attributed to phase variation of these genes, which results in variability in both the level and location of MeOPN modifications [88].

The recent investigations have thus highlighted the complexity and high variability of the CPS biosynthesis, and provided insights into the enzymes central to this biosynthetic pathway, which may in future be considered as targets for anti-*C. jejuni* therapeutic applications.

### Autoinducer-2 and quorum sensing in *C. jejuni*

Collective behaviours driven by quorum sensing (QS) play a major role in *C. jejuni* virulence and persistence within the environment. QS is a density-dependent gene regulation achieved as a result of secretion and sensing of signalling molecules termed autoinducers. In *C. jejuni*, many virulence genes as well as lifestyle modification such as biofilm formation, are regulated in a QS-dependent manner.

QS in *C. jejuni* relies upon *luxS* [89], which encodes an S-ribosylhomocysteine lyase responsible for the hydrolysis S-ribosylhomocysteine to homocysteine, with 4,5-dihydroxy-2,3-pentadione (DPD) generated as a by-product [89]. DPD derivatives spontaneously rearrange to several forms, including the quorum signal autoinducer-2 (AI-2) [90]. Increased cell density is associated with increased *luxS* expression and AI-2 production in *C. jejuni* [91]. However, the mechanism of AI-2 export remains unclear. While it has been shown by membrane permeability assessments that AI-2 molecules are hydrophilic [92] and thus, relatively membrane-impermeable, their mode of export has not been characterised in *C. jejuni*, and there is still limited understanding of the function of the AI-2 exporters identified in other bacterial species [92].

Recently, a small noncoding RNA, CjNC110, has been identified downstream of *luxS* in *C. jejuni* [93]. CjNC110-knockout mutants showed significantly increased accumulation of intracellular AI-2 compared to WT, while extracellular AI-2 levels were reduced. This suggested that the CjNC110 RNA modulates AI-2 detection and export [93]. However, the role of this regulatory RNA in QS regulation and AI-2 export requires further investigation.

The precise mechanism of AI-2 uptake or sensing by *C. jejuni* has likewise not been fully elucidated. Comparative genomics has not revealed the presence in *C. jejuni* of any homologues of either the *lsr*-encoded ABC transporter, which imports AI-2 in enteric bacteria such as *Salmonella* typhimurium, or the AI-2 sensing two-component regulatory system LuxPQ, which transmits the signal generated from AI-2 binding in *Vibrio* species [94]. Despite this, in an AI-2 uptake assay assessing *E. coli* as well as the WT strain and *luxS* mutants of *C. jejuni* in the presence of exogenous AI-2 [95], exogenous AI-2 levels decreased over six hours in *E. coli* culture, but not in cultures of WT or Δ*luxS* strains of *C. jejuni* [95]. AI-2 mediated activity was still detected in *C. jejuni* Δ*luxS* mutant cells, suggesting that exogenous AI-2 molecules were not imported, but rather, interacted with a two-component signalling system to exert their effect [95].

### AI-2 in *C. jejuni *– regulation of motility, virulence and biofilm formation

LuxS has been implicated in diverse aspects of *C. jejuni* virulence. A specific role for *luxS* has been shown in invasion, as *luxS*-deficient mutants of *C. jejuni* NCTC 11168 displayed reduced capacity for invasion of INT407 epithelial cells compared to WT [96]. This may have been due to QS-mediated upregulation of secreted Cia proteins that facilitate host cell actin reorganisation required for host cell invasion [50]. Given that Cia proteins are secreted via the flagellar T3SS, QS-mediated upregulation of flagellins may also indirectly support invasion [50, 96].

DNA microarrays also showed that *luxS*-deletion mutants expressed significantly lower levels of *ahpC* and *tpx* (encoding for alkyl hydroperoxide reductase and thiol peroxidase, respectively) compared to WT *C. jejuni* 81–176, and displayed a correspondingly lower resistance to H_2_O_2_ and cumene hydroperoxide [97]. This indicates that QS may be responsible for upregulation of certain genes associated with the oxidative stress response [97].

**Fig. 3:**
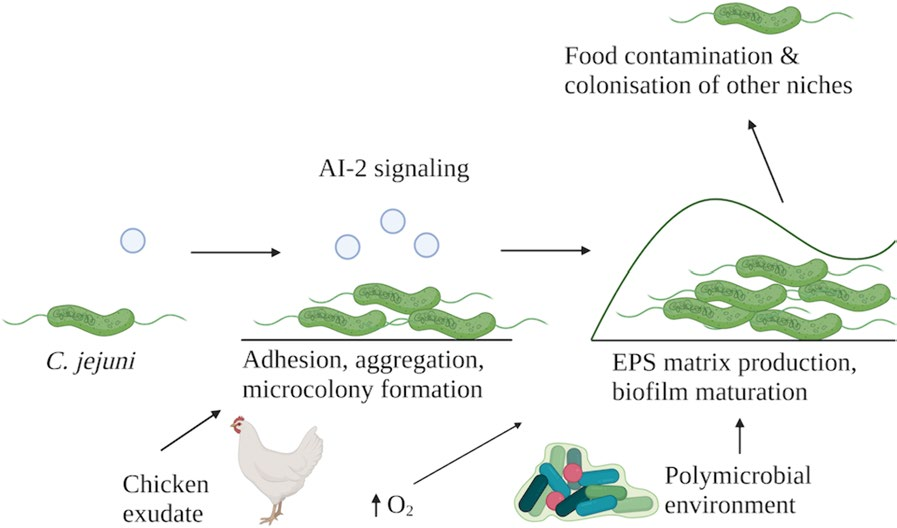
An overview of the steps and factors involved in *C. jejuni* biofilm formation. *C. jejuni* biofilm formation is initiated by adhesion, aggregation and microcolony formation, which is followed by biofilm maturation and EPS matrix production. Biofilm formation in *C. jejuni* is a process regulated by autoinducer 2-mediated gene expression, and has been shown to be enhanced by environmental factors including the presence of chicken exudate, environmental oxygen levels and the presence of other bacterial species in the environment such as *P. aeruginosa.* The presence of a *C. jejuni* biofilm enables the release of planktonic cells into the environment, facilitating their further contamination of surfaces in the environment of a poultry processing facility, or enabling colonisation of other niches within a host environment

A biofilm lifestyle is a major persistence strategy of *C. jejuni*, which enables it to exist as an adherent bacterial community, encased in an extracellular polymeric substance matrix. Cells within a biofilm display altered gene expression and metabolic states, enabling them to remain protected from external stresses, such as antibiotics, host immune responses, and, during persistence in food processing facilities, from atmospheric oxygen levels, thus promoting persistence [98]. Biofilms develop through initial adhesion of bacterial cells to a surface and aggregation with other bacterial cells, secretion of extracellular polymeric substances, and biofilm maturation [99]. An overview of *C. jejuni* biofilm formation and its relation to its persistence and spread is demonstrated in Fig. 3.

QS has been shown to play a role in biofilm formation in *C. jejuni*. Mutants lacking LuxS displayed significantly reduced biofilm formation compared to the WT 81–176 strain [100]. However, the fact that they could still form biofilms suggests the involvement of additional alternative regulatory factors in controlling the expression of biofilm formation genes [101].

One of the mechanisms by which QS aids biofilm formation is the regulation of genes involved in flagellation and motility. LuxS-mediated QS has been shown to modulate *C. jejuni* motility in soft agar [96]. This aligns with prior experiments where *luxS*-deletion mutants displayed decreased transcription of *flaA* [102], which encodes one of the two flagellins [103]. Importantly, while flagellation [104] and flagellin glycosylation [105, 106] are essential for biofilm formation [104], motility has also been shown to promote biofilm formation [99], and the biofilm community was shown to harbour both sessile and motile *C. jejuni* populations [107]. Furthermore, a link between *luxS* and regulation of chemotaxis towards amino acids and organic acids has been demonstrated [108], while a separate study established that essential chemotaxis genes (*cheA*, *cheY*, *cheV* and *cheW* ) played a significant role in biofilm formation, as their deletion resulted in altered biofilm architecture [15]. Collectively, these studies highlight the complex interplay of the AI-2 quorum sensing system, flagellation, motility and chemotaxis in *C. jejuni* biofilm formation.

### Biofilm formation and persistence in the environment and human host

*C. jejuni* strains have been shown to form biofilm on surfaces associated with poultry processing facilities, including stainless steel, and biofilms are believed to support the transmission and persistence of *C. jejuni* through the food processing chain [109, 110]. *C. jejuni* can form both single-species biofilms, and polymicrobial biofilms with common environmental organisms including *Pseudomonas aeruginosa* [111]. *P. aeruginosa* has been shown to enhance the biofilm formation of *C. jejuni* by generating a local microaerophilic environment favoured by *C. jejuni* [112].

Within poultry production facilities, lipid- and protein-rich exudate from slaughtered chicken builds up on equipment surfaces [109, 113]. The exudate, that is often contaminated with the contents of the gastrointestinal tract of chickens [114], was shown to significantly enhance *C. jejuni* biofilm formation compared to Brucella broth [114]. Exposure to atmospheric oxygen also significantly upregulated biofilm formation of *C. jejuni* strains NCTC 11168 and 81–176 [107] compared to growth under microaerobic conditions [115].

A more intricate, strain-dependent relationship between biofilm formation capacity, the material of attachment surface and atmospheric oxygen levels has also been shown recently. Hyperaerotolerant strains of *C. jejuni* exhibited significantly increased biofilm formation on polystyrene surfaces compared to aerotolerant strains, particularly in aerobic conditions. Yet, it was the aerotolerant isolates that demonstrated a significantly higher potential for biofilm formation on stainless steel, regardless of atmospheric oxygen levels. These results indicate that the enhanced ability of oxygen-tolerant strains to form biofilms depends on the surface material [116].

These findings suggest that poultry processing facilities provide an environment with a high potential for formation of *C. jejuni* biofilms, and therefore, act as sources of contamination for poultry that is being packaged [117]. This contamination is compounded by the release of planktonic cells from mature biofilms [118], which further drives the cycle of poultry contamination and spread of *C. jejuni*.

While many studies have explored the role of biofilms in allowing *C. jejuni* persistence in the environment, it is widely known that biofilms promote persistence of diverse enteric pathogens in the human gastrointestinal tract [112]. Unsurprisingly, scanning electron microscopy (SEM) has shown that *C. jejuni* 81–176 forms microcolonies, the biofilm precursor, within 1–2 h after in vitro infection of human ileal tissue [101]. Microcolony formation involves attachment of cells to one another and adherence to the mucus lining the intestine. By 3–4 h after infection in vitro, SEM demonstrated the formation of widespread biofilms [101]. Since biofilm formation is associated with changes in metabolic activity and increased resistance to reactive oxygen species in *C. jejuni* [119], biofilms may support persistence in the human host by shielding cells from host immune responses [101], although the role of biofilms in *C. jejuni* infection in humans is an area of study warranting further exploration.

### Cytolethal distending toxin and inflammation

The pro-inflammatory response induced by *C. jejuni* contributes to the symptoms of mucosal inflammation observed in campylobacteriosis [120]. *C. jejuni* has been shown to elicit a pro-inflammatory cytokine response by human monocytes [121] and intestinal epithelial cells [122], which involves NF-κB stimulation and release of pro-inflammatory cytokines and chemokines such as interleukin 8 (IL-8), which recruits neutrophils to the site of infection [122]. One of the key causes of the pro-inflammatory response is the cytolethal distending toxin (CDT). CDT is considered a major virulence factor of *C. jejuni*, and 74–100% of *C. jejuni* isolates possess the CDT-encoding genes (*cdt*ABC) [103]. Recently, the roles of CDT in inflammation have been more extensively explored, providing novel insights into its contribution to the pathogenesis.

CDT is secreted by *C. jejuni* via outer membrane vesicles [65]. One way in which CDT contributes to enterocolitis is by causing local acute inflammation of the intestine during infection. As demonstrated in colonic epithelial cells, CDT induces the secretion of IL-8 via the activation of NF-κB [123]. CDT also causes host cells to arrest in the G2/M phase of the cell cycle, and leads to cytoplasmic distension [124].

In order for CDT to be functional, all three of its protein subunits (CdtA, CdtB, and CdtC) are required [125]. The subunits exhibit cellular toxic activity only when a tripartite holotoxin is formed, where the role of the CdtA/CdtC heterodimer is to deliver the enzymatically active CdtB subunit into target epithelial cells [124]. It is the enzymatic activity of CdtB that underpins the toxic effects of CDT. CdtB has been known for a while to function as type I deoxyribonuclease (DNAse I) that cleaves double stranded DNA within host cells [126, 127]. However, a recent study has led to a novel paradigm for the CdtB function distinct from its DNAse I activity: CdtB exhibits phosphatidylinositol 3-4-5 trisphosphate (PIP3) phosphatase activity in lymphocytes, which blocks PI-3 K signaling in lymphocytes, and results in toxin-induced cell cycle arrest and apoptosis [128]. In addition, blockade of the PI-3 K signaling has a pro-inflammatory effect, inducing the production of IL-1β, TNFα and IL-6 by macrophages [129].

Moreover, CDT has been shown to induce pyroptosis, a form of pro-inflammatory programmed cell death during which the cell membrane ruptures, releasing cell contents into the microenvironment of human colonic epithelial cells in a dose- and time-dependant manner. Inflammation is central in pyroptosis, which results in rupture of the cells and associated release of pro-inflammatory cytokines [130]. This form of programmed cell death was induced via the ROS/caspase-9/caspase-3/gasdermin E (GSDME) pathway [131]. Importantly, the CDT-mediated apoptosis of lymphocytes could result in *C. jejuni* persistence, whereas pyroptosis of intestinal epithelial cells is likely to cause epithelial barrier dysfunction, further contributing to symptoms of infection, and potentially, to *C. jejuni* persistence. Indeed, CDT has been shown to contribute to long-term colonisation by *C. jejuni* [132].

It has been previously shown that the TlyA-mediated methylation of nucleotide C1920 in 23 S rRNA is important for virulence, including adherence [133], biofilm formation and motility [134]. A recent study employing a proteomics approach has added CDT to the list of virulence factors controlled at the expression level via this mechanism [135].

### Post-infection sequelae – Guillain-Barre syndrome

*C. jejuni* is associated with many long-term sequelae, including dysbiosis, irritable bowel syndrome (IBS), and Guillain-Barré syndrome (GBS), which cause further disease burden even following the resolution of acute infection. The most well-known sequela of *C. jejuni* infection is GBS. GBS is a condition characterised by acute, flaccid, neuromuscular paralysis [136]. Inflammation-mediated damage to myelin sheaths observed in the acute inflammatory demyelinating polyneuropathy (AIDP) subtype of GBS and/or damage to the axon observed in the acute motor axonal neuropathy (AMAN) subtype of GBS inhibits the transmission of nerve signals throughout the body, causing dysfunction of the peripheral nervous system and paralysis [137]. Infections are the most common trigger of GBS, preceding 75% of all cases [138]. *C. jejuni* infections have been found to cause ~ 30% of all GBS cases, including both the AMAN and AIDP subtypes, with differing frequencies reported for different patient populations [139].

IgG anti-ganglioside antibodies are elevated in 41–85% of patients with *C. jejuni*-induced GBS [140]. Gangliosides are acidic glycosphingolipids that have many functions, including in maintaining neurophysiological balance, cell signalling, and cell-cell communication [140, 141]. They are distributed throughout the body and are particularly abundant in the brain and peripheral nervous system [141]. Gangliosides are present in the myelin sheath of nerve cells – an insulating layer that enables the efficient transfer of electrical signals, as well as, to a greater extent, in the axonal membrane. The loss or modification of gangliosides is associated with a range of neurological diseases, including GBS [137].

Structurally, gangliosides are composed of a hydrophobic ceramide lipid tail attached to a glycan headgroup that contains one or more sialic acid residues [142]. *C. jejuni* induces the development of GBS via the mechanism of host ganglioside mimicry that involves *C. jejuni* lipooligosaccharide (LOS). LOS is a C. *jejuni* cell membrane component comprising a hydrophobic lipid A anchor and an oligosaccharide with a conserved inner and variable outer core – but lacking the O-antigen characteristic of LPS [143]. LOS and gangliosides have similar structure, which enables host antibodies that bind LOS to also bind to gangliosides, eliciting an autoimmune response [144]. Indeed, it was observed that rabbits immunised with bovine gangliosides developed anti-GM1 antibodies and presented with GBS symptoms [145]. The anti-LOS antibodies cross-react with gangliosides, enabling macrophages, the complement system and other immune mediators to bind to the autoantibody and trigger axonal degradation and potentially contribute to demyelination of peripheral nerves [137, 146]. While *C. jejuni* LOS induced anti-GM1 antibodies in a rabbit model, LOS from *E. coli* or *Salmonella minnesota* failed to do so [147]. This suggests that it is the oligosaccharide portion of the *C. jejuni* LOS that mimics the structure of GM1 gangliosides, rather than its conserved lipid A moiety.

Damage in GBS is hypothesised to be primarily immunopathological, with almost no influence by *C. jejuni* directly. The complement system may cause neuronal demyelination and axonal degeneration through membrane attack complexes. The complement system can encourage inflammation by releasing anaphylatoxins such as C3a and C5a [148], and encourage the infiltration of macrophages. The macrophages produce pro-inflammatory molecules, such as tumour necrosis factor-α (TNFα) and free radicals, further encouraging host damage. Moreover, macrophages can also cause damage to nerve cells by myelin phagocytosis [148]. Furthermore, the release pro-inflammatory cytokines by CD4 + T cells that infiltrate the blood nerve barrier may enhance the destructive activity of macrophages [148]. All these immunopathological mechanisms contribute to the axonal degradation and demyelination of peripheral nerves that cause GBS symptoms.

Treatment of GBS to date involved plasma exchange (plasmapheresis), which removes inflammatory components from the blood, and intravenous immunoglobulin administration, which dampens the immune response and reduces the autoantibody levels [149]. Administration of proteases and monoclonal antibodies are novel approaches to GBS treatment currently being investigated [150]. However, gaining a deeper understanding of post-*C. jejuni* GBS may lead to more optimal treatment approaches.

### Post-infectious IBS

**Fig. 4:**
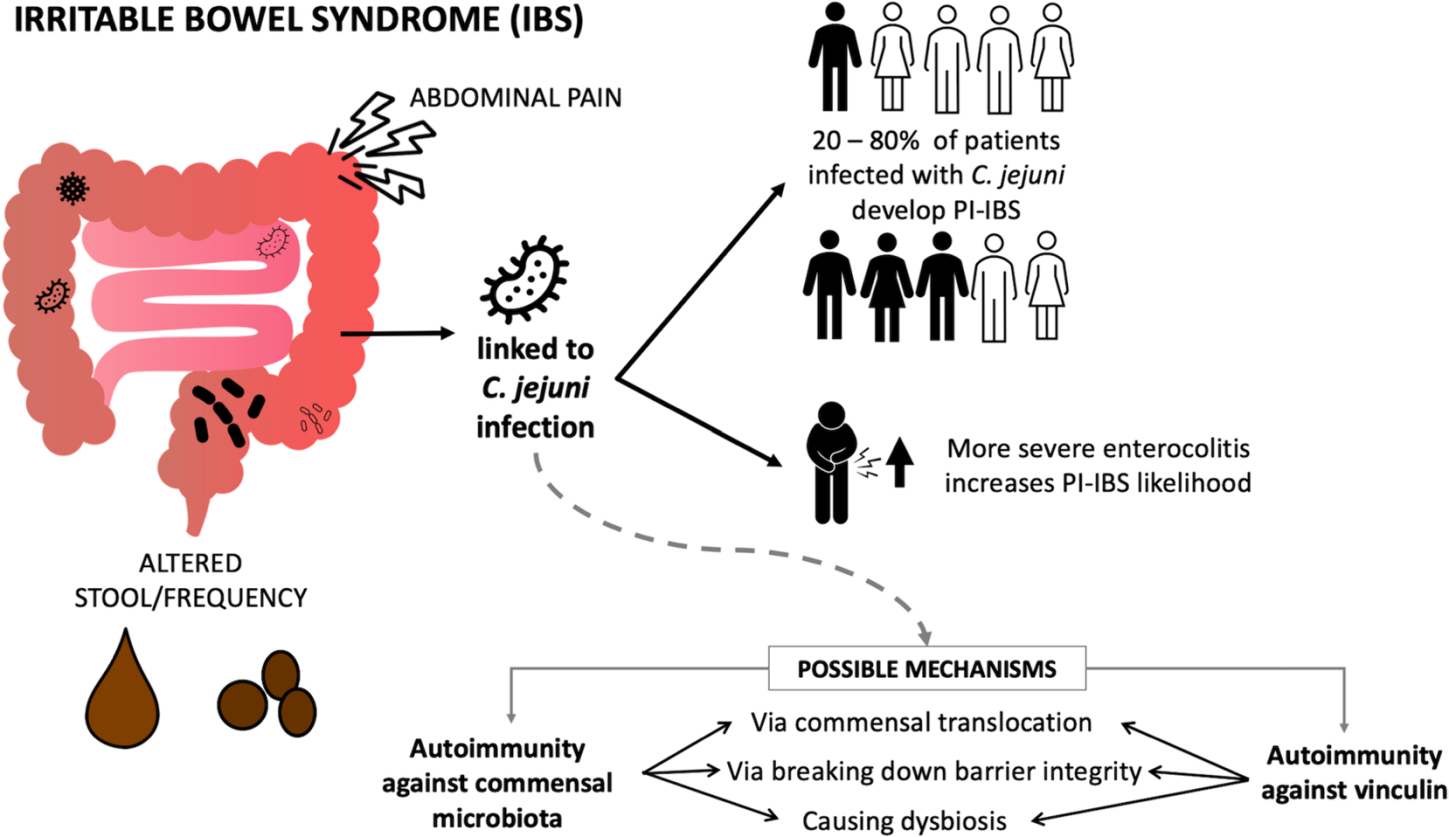
PI-IBS, a long-term sequela of *C. jejuni* infection observed in patients with *C. jejuni* enterocolitis. Severity of enterocolitis has been linked to increased likelihood of PI-IBS development, and potential mechanisms of PI-IBS development include the development of autoimmunity against commensal bacteria, as well as autoimmunity against the host protein vinculin. These mechanisms subsequently result in the *C. jejuni*-mediated translocation of commensal bacteria, reduction in membrane integrity, and dysbiosis

Irritable bowel syndrome (IBS) is another potential long-term sequela of *C. jejuni* infection. Key symptoms include abdominal pain and alteration in frequency and/or form of stool, with varying severity [151]. Post-infectious IBS (PI-IBS) is a subset of IBS that can develop after infection, and the likelihood of PI-IBS development has been linked to the severity of enterocolitis symptoms during infection [151]. The estimated rate of PI-IBS development after *C. jejuni* infection is 20-80% [151, 152], and likely depends on the virulence of *C. jejuni* strains (Fig. 4).

Indeed, the link between certain *C. jejuni* clonal complexes and virulence factors to PI-IBS isolates has been explored [153], and ST-922 clonal complex lineages were identified to be the most over-represented in PI-IBS patient isolates [153]. Surprisingly, the PI-IBS isolates did not have significant differences in the prevalence of known virulence genes compared to controls. However, a genome-wide association study found that *C. jejuni* strains harbouring variants in genes involved in stress response and in biosynthetic pathways for biotin, purines and isoprenoids were most significantly associated with PI-IBS. Moreover, PI-IBS patient isolates displayed greater adhesion to T84 epithelial cells and greater intracellular invasion [153].

### PI-IBS caused by autoimmunity against translocated commensals

PI-IBS development is complex and multifaceted because it is influenced by host genetic factors, microbiota and bacterial virulence factors. Acute *C. jejuni* infection can lead to gastrointestinal dysfunction that causes IBS-like symptoms in the short-term; however, long-term PI-IBS is not associated with continued *C. jejuni* infection [154–156]. Rather, autoimmunity and prolonged dysbiosis are thought to be key mechanisms for continued IBS symptoms post *C. jejuni* infection.

One potential mechanism of pathogenesis is *C. jejuni* inducing an immune response targeting commensal bacteria. Indeed, *C. jejuni* is known to promote translocation of non-pathogenic *E. coli* across the intestinal barrier [157]. The resulting inflammation and immune response may disrupt normal gut function and microbiota, and cause symptoms that persist after infection clearance.

In addition to facilitating translocation of members of the commensal microbiota to the basolateral surface of the epithelium, *C. jejuni* exposure was found to be associated with transcriptional changes in non-invasive *E. coli* resulting in its upregulation of virulence genes associated with the flagellum, adhesins, biofilm, hemolysin, and stress resistance [158]. This enhanced virulence could potentially trigger an inappropriate immune response against the commensals, possibly leading to continuous gut inflammation and immunity against the microbiota, eventually causing a disruption to its composition.

Human studies have also supported an association between *C. jejuni* and dysbiosis. Patients who developed PI-IBS after *C. jejuni* infection had a more disturbed microbiota composition than those who fully recovered from the initial acute bacterial infection [156]. Another study observed that children with asymptomatic *C. jejuni* infection had reduced bacterial diversity and richness in their gut microbiota, with a negative impact on child growth [159].

### PI-IBS caused by autoimmunity against vinculin

CdtB, described earlier in this review, has been linked to the development of PI-IBS. CdtB is associated with PI-IBS symptoms by triggering autoimmunity against host vinculin, a focal adhesion protein. As a component of membrane-associated complexes that mediate cell-to-cell and cell-to-matrix adhesion [160], vinculin has roles in cell migration, cell adhesion, and gut motility [160, 161]. Rats exposed to CdtB alone (via injection) developed IBS-like phenotypes, with anti-CdtB antibodies and autoantibodies for vinculin accumulating in the gut [162]. Moreover, reduced vinculin levels were observed in the vaccinated mice. It is thought that antibodies generated against CdtB cross-react with vinculin. A reduction in vinculin levels is proposed to destabilise adherens junctions in the gut epithelium, contributing to disrupted gut function, and subsequently, diarrhea and eventual microbial dysbiosis [162]. Indeed, 4 months after being infected and cleared of CdtB-positive *C. jejuni*, rats continued to show IBS-like symptoms, including chronic altered bowel pattern, rectal inflammation and reduced deep muscular plexus interstitial cells of Cajal (ICC-DMP) - “intestinal pacemakers” that are required for normal intestinal motility [163]. Damaged ICC cells following *C. jejuni* infection are associated with disrupted neural control of gastrointestinal contractions, leading to disorders such as IBS [155]. These findings are corroborated by recent investigations, which have identified IBS patients to have higher levels of anti-CdtB and anti-vinculin antibodies compared to healthy controls [164].

### Vaccines

**Fig. 5:**
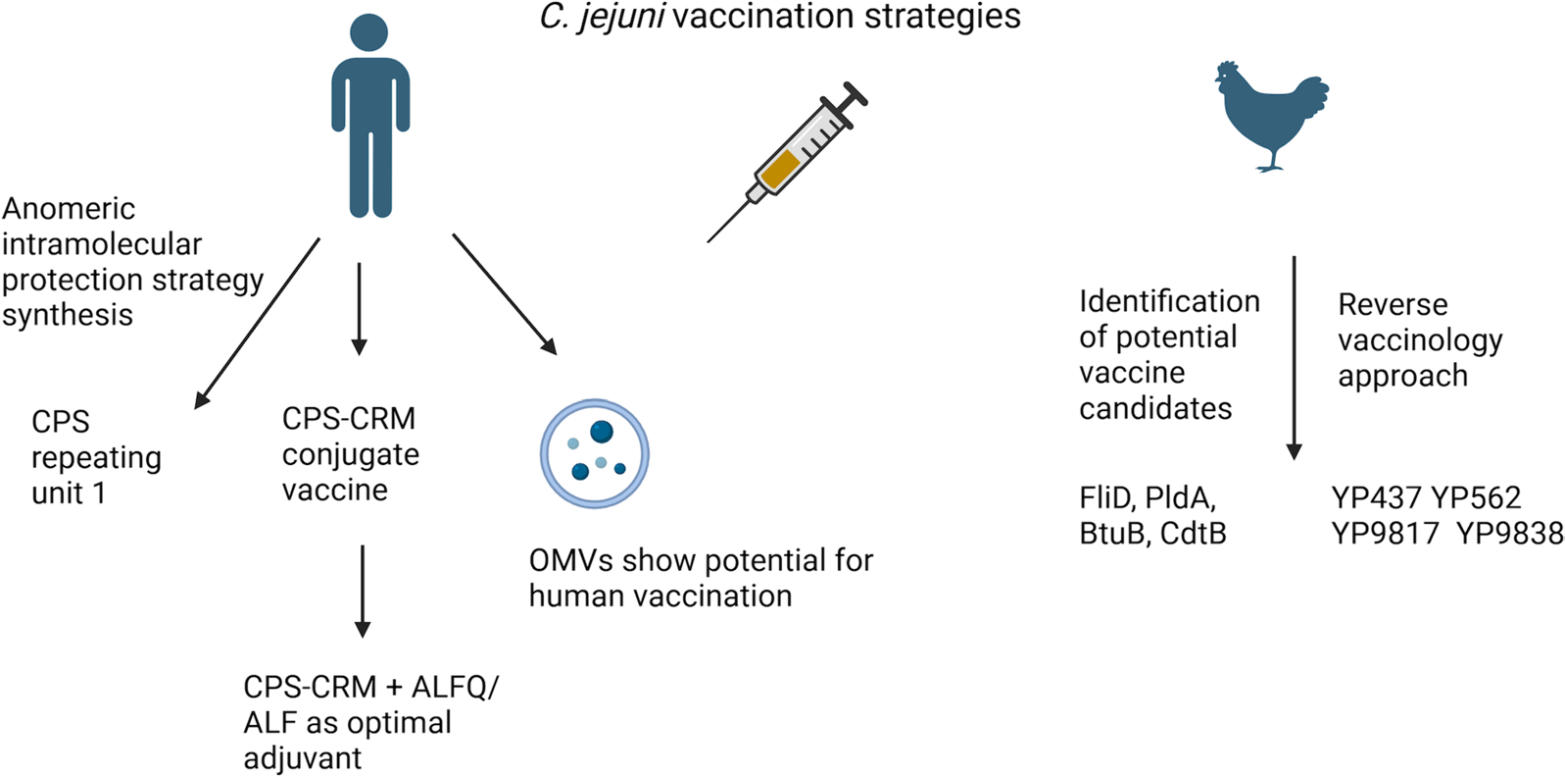
A summary of the progress in vaccine development for *C. jejuni*. Development of a CPS-based vaccine for human use required synthesis of CPS repeating unit 1 and involved trials of a conjugate CPS-CRM vaccine. The CPS-CRM vaccine has shown promise in animal models, with ALFQ as the preferred adjuvant. OMVs have also recently emerged as a suitable vaccine candidate, showing promise in a mouse model. However, further safety testing is required due to the presence of LOS within the OMVs. Efforts have also been directed towards development of a *C. jejuni* vaccine for use in chickens to limit *C. jejuni* colonisation and subsequent transmission to humans. This approach lead to the identification of conserved proteins FliD, PldA, BtuB and CdtB as vaccine antigen candidates. Finally, a reverse vaccinology approach, which includes bioinformatics analysis of sequence conservation, subcellular localisation and predicted immunogenicity, has pinpointed 4 new vaccine candidates, which showed promise in both mouse and chicken trials

Despite the significant impact of *C. jejuni* infections on public health, including the long-term sequelae, there are currently no approved *C. jejuni* vaccines for either human or poultry use. Due to the presence of LOS on the *C. jejuni* cell surface and its potential to trigger autoimmunity resulting in GBS, administration of live or attenuated whole-pathogen vaccines for humans is not desirable. Subunit vaccines based on selected antigens appear to be a more appropriate choice [165]. Recent progress in the vaccine development field is overviewed in Fig. 5.

The CPS of *C. jejuni* has been the main target of interest in vaccine development in recent years [166, 167]. The main challenges are the relatively low immunogenicity of CPS fragments and the requirement of using a conjugate vaccine in order to elicit a significant level of immune response [167]. Importantly, the unique MeOPN moiety on the *C. jejuni* CPS [168] has been demonstrated to be required for an immunological response, and thus *C. jejuni* strains expressing MeOPN transferases in the “on” phase have been recommended for the CPS production [167, 168]. A chemical synthesis approach using an intramolecular anomeric protection strategy (iMAP) has also been evaluated for the production of the CPS repeating unit 1 (tetrasaccharide), and is believed to protect reactive sites such as the MeOPN moiety from unwanted reactions, which can occur during the conjugation process [167]. This approach enabled synthesis of the NCTC 11168 CPS repeating unit 1 with a complete MeOPN moiety. The use of unit 1 as a potential vaccine candidate is currently under further investigation [167].

Another CPS-based vaccine study performed by Monteiro et al. used the HS23 and HS36 serotype complex from the *C. jejuni* strain 81–176 and the HS4 serotype complex from the CG8486 strain [166]. The complexes were linked to the non-toxic variant of diphtheria toxin CRM_197_ forming a CPS-CRM conjugate with a stoichiometry of 2–5 CPS moieties per 1 CRM_197_ [166]. Subcutaneous administration of these glycoprotein conjugates in mice elicited a robust immune response characterised by an increase in CPS-specific IgG, which was maintained for up to 26 weeks following vaccination. This vaccination strategy also significantly reduced disease severity when the mice were subsequently challenged intranasally with homologous strains of *C. jejuni* [166]. In addition, the conjugated vaccine for 81–176 conferred full protection against *C. jejuni*-associated diarrhea in the New World monkeys [166]. However, the conjugate CPS vaccine was not immunogenic in humans, both with and without the use of alum as an adjuvant [168]. To overcome this limitation, a subsequent study performed by Ramakrishan et al. investigated the immunogenicity and protective efficacy of the conjugate vaccine when administered with novel liposome-based adjuvants [169], the Army Liposome Formulation (ALF) containing synthetic monophosphoryl Lipid A and ALFQ, which contained ALF and QS-21, a purified plant extract from *Quillaja saponaria* that is commonly used as a vaccine adjuvant. Compared to CRM-CPS alone, the ALF and ALFQ adjuvant vaccines elicited a bias towards a Th1-mediated production of IgG2b and IgG2c antibodies against CPS in a mouse immunisation model [169]. Notably, the ALFQ + CPS-CRM vaccination appeared to enhance T cell responses, leading to the increased production of Th1, Th2, and IL-17 cytokines when compared to CPS-CRM alone or CPS-CRM + ALF [169]. Furthermore, immunisation with ALFQ + CPS-CRM resulted in higher levels of serum bactericidal antibodies, and New World monkeys vaccinated with ALFQ + CPS-CRM showed enhanced protection against diarrhoea upon *C. jejuni* challenge, compared to CPS-CRM + alum or CPS-CRM + ALF, indicating the promising results of ALFQ use as an adjuvant in a *C. jejuni* vaccine [169].

Importantly, immunization of chickens is viewed as a potential pathway to reduce chicken colonisation by *C. jejuni*, and thus limit its spread to humans. Antibodies against the flagellar capping protein FliD have been recently found in all assessed chicken sera, opening avenues for the evaluation of this protein as a vaccine candidate [170]. Other studies of note evaluated the following antigenic proteins as potential vaccine candidates: PldA (phospholipase A), which plays a major role in hemolytic activity and assists in host colonization [171]; BtuB (TonB dependent B12 transporter), an OMP essential for transport of the vitamin B12 [172, 173]; and CdtB [174], the CDT subunit present in over 90% of *C. jejuni* poultry isolates [175, 176]. A study based on the reverse vaccinology approach identified PldA, BtuB and CdtB as suitable candidates to be used in a *C. jejuni* subunit vaccine [175]. Another study utilising a bioinformatics approach, which involved selection of proteins based on cellular localisation, number of transmembrane helices, predicted antigenicity and conservation across *C. jejuni* strains, identified 3 extracellular flagellar proteins and 11 OMPs as potential vaccine antigens [177]. Subsequent screening for protective immunity revealed that four of these - OMPs YP437, YP9817 and YP9838, and flagellar protein FlgL (YP562) - substantially reduced *C. jejuni* cecal load in chickens [178]. However, limited results reproducibility in that study warrants further investigation.

### Future directions

The rapidly evolving field of *C. jejuni* research is revealing a multitude of diverse mechanisms of *C. jejuni* pathogenesis which enable it to colonise human and animal hosts with varying disease outcomes and to persist in the environment of poultry processing facilities. This review has explored the major advances in this field of research and proposed avenues for future investigation as summarised below.

#### Chemotaxis

Little is known about how a multitude of attractant and/or repellent signals within a chemically complex in vivo environment are integrated by more than 10 different Tlps in *C. jejuni*, and how they interrelate with other virulence mechanisms. A better understanding of this system as a whole may lead to an understanding of how these sensing mechanisms can be harnessed for the design of therapeutic compounds. For example, this understanding could aid in reducing colonisation in chickens and consequently reduce transmission to humans.

#### CPS capsule

The polysaccharide capsule appears to play a significant role in colonisation, displaying high structural diversity and phase variability, which facilitates immune evasion. An improved understanding of the enzymes responsible for creating this diversity could help identify compounds that inhibit the CPS biosynthesis pathways, thereby reducing adhesion and invasion. Recent studies have highlighted the additional roles of the heptose moiety of the CPS, including boosting intracellular survival and lowering the levels of macrophage-generated NO. Deeper understanding of the structure-function relationship in the CPS may also help identify the most effective immunisation strategies for CPS-based vaccines.

#### QS and biofilms

QS-driven behaviour and biofilm formation in *C. jejuni* have been extensively studied in the context of environmental survival. However, little is known about the AI-2 secretion and uptake pathways. Harnessing the AI-2 system to prevent biofilm formation would enable better management of *C. jejuni* spread in the poultry-processing facilities. Moreover, a significant gap in biofilm research lies in understanding biofilm formation in the human intestine during infection, and the role these structures may play in shielding cells from the immune response, as well as promoting longer-term persistence.

#### T3SS and T6SS

Recent in silico methods have identified many putative T3SS and T6SS effector proteins. However, their specific functions and significance in disease, as well as their roles within the T3SS and T6SS mechanisms, have yet to be fully determined. Despite its role in adhesion and invasion, the importance of the T6SS in *C. jejuni* remains somewhat enigmatic, as only up to 20% of isolates have been found to harbour it, and its presence has not been associated with more severe disease. Further studies are required to explore its potential impact on disease outcomes, its influence on the microbiome, and its contribution to survival mechanisms in the environment.

#### OMVs

OMVs have emerged as key players in *C. jejuni* interactions with the host. It is intriguing that the composition of the OMV proteome is affected by the changes in the environmental conditions. Further comprehensive in vivo studies are essential to uncover the roles of the OMV proteome in transmission, colonisation and disease progression, as well as to identify the specific contributions of individual protein components to the pathogenesis.

#### Vaccines and therapeutics

Significant progress has been made in *C. jejuni* vaccinology, which including advancements in the production of a CPS-based vaccine for human use. While efforts are underway to utilise the anomeric molecular protection strategy for CPS synthesis in vaccine production, and a CPS-CRM conjugate vaccine has already shown potential in animal models, clinical trials are necessary to assess efficacy in humans. Antigen candidates have been identified for a vaccine for chickens, but further animal studies, including avian model studies, are needed to achieve more consistent results and efficacy. OMVs show promise as a novel vaccine candidate for use in humans. However, further safety assessments are necessary, particularity due to the presence of LOS.

A growing understanding of the virulence mechanisms of *C. jejuni* opens avenues for the discovery of therapeutic molecules that could interfere with *C. jejuni* colonisation and pathogenesis. These molecules may include those that block pathways involved in chemotaxis, biofilm formation, adhesion and invasion, as illustrated in Fig. 1. These strategies hold significant promise and merit further exploration in the field of *C. jejuni* research.

## Conclusions

*C. jejuni* remains a leading cause of gastroenteritis. The potential short- and long-term sequelae, including Guillain-Barre syndrome, highlight its importance as a pathogen that detrimentally impacts human quality of life. This review has summarised recent progress in understanding the molecular mechanisms involved in colonisation, invasion of cells, collective quorum sensing-mediated behaviours, persistence, and the basis of *C. jejuni*-triggered autoimmunity. It has also highlighted the gaps to be addressed by future research.

## Data Availability

Not applicable.

## Abbreviations

IBS: Irritable bowel syndrome
GBS: Guillain-Barré syndrome
T3SS: Type III secretion system
Tlp: Transducer-like protein
HtrA: High-temperature requirement protein A
CCV: *Campylobacter*-containing vacuole
WT: Wild-type
T6SS: Type VI secretions system
Cia: *Campylobacter* invasion antigen
IL-8: Interleukin-8
Hcp: Hemolysin co-regulated protein
OMV: Outer membrane vesicle
OMP: Outer membrane protein
CPS: Capsular polysaccharide
HS: Heat-stable
NO: Nitrogen oxide
PLP: Pyridoxal-5′-phosphate
MeOPN: O-methyl phosphoramidate
QS: Quorum sensing
DPD: 4,5-Dihydroxy-2,3-pentadione
AI-2: Autoinducer-2
SEM: Scanning electron microscopy
CDT: Cytolethal distending toxin
DNAse I: Type I deoxyribonuclease
PIP3: 3-4-5 trisphosphate
AIDP: Acute inflammatory demyelinating polyneuropathy
AMAN: Acute motor axonal neuropathy
LOS: Lipooligosaccharide
TNFα: Tumour necrosis factor-α
PI-IBS: Post-infectious IBS
ICC-DMP: Deep muscular plexus interstitial cells of Cajal
iMAP: Intramolecular anomeric protection
ALF: Army Liposome Formulation
PldA: Phospholipase A
BtuB: TonB dependent B12 transporter
